# Influence of species and anatomical location on chondrocyte expansion

**DOI:** 10.1186/1471-2474-6-23

**Published:** 2005-05-17

**Authors:** Margarete K Akens, Mark B Hurtig

**Affiliations:** 1 Comparative Orthopaedic Research Laboratory, Dept. of Clinical Studies, University of Guelph, Guelph, Ontario, N1G 2W1, Canada

## Abstract

**Background:**

Bovine articular cartilage is often used to study chondrocytes *in vitro*. It is difficult to correlate *in vitro *studies using bovine chondrocytes with *in vivo *studies using other species such as rabbits and sheep. The aim of this investigation was to study the effect of species, anatomical location and exogenous growth factors on chondrocyte proliferation *in vitro*.

**Methods:**

Equine (EQ), bovine (BO) and ovine (OV) articular chondrocytes from metacarpophalangeal (fetlock (F)), shoulder (S) and knee (K) joints were cultured in tissue culture flasks. Growth factors (rh-FGFb: 10 ng/ml; rh-TGFβ: 5 ng/ml) were added to the cultures at days 2 and 4. On day 6, cells were counted and flow cytometry analysis was performed to determine cell size and granularity. A three factor ANOVA with paired Tukey's correction was used for statistical analysis.

**Results:**

After 6 days in culture, cell numbers had increased in control groups of EQ-F, OV-S, OV-F and BO-F chondrocytes. The addition of rh-FGFb led to the highest increase in cell numbers in the BO-F, followed by EQ-F and OV-S chondrocytes. The addition of rh-TGFβ increased cell numbers in EQ-S and EQ-F chondrocytes, but showed nearly no effect on EQ-K, OV-K, OV-S, OV-F and BO-F chondrocytes. There was an overall difference with the addition of growth factors between the different species and joints.

**Conclusion:**

Different proliferation profiles of chondrocytes from the various joints were found. Therefore, we recommend performing *in vitro *studies using the species and site where subsequent *in vivo *studies are planned.

## Background

*In vitro *studies regarding chondrocyte metabolism and expansion are often performed using bovine chondrocytes [1-3]. These chondrocytes are harvested from the metacarpophalangeal (fetlock) joint of slaughter-age cattle (18 months old or younger), since the distal limb is not used for meat production. However, *in vivo *animal studies not only tend to be performed in other animals, such as rabbits [4-9] and sheep [10-14], but also tend to use a different joint. Rather than the fetlock joint used in *in vitro *studies, the knee joint is used for *in vivo *animal studies [11,12], since it is frequently affected by osteoarthritis in humans [15,16].

Animal models of osteoarthritis are used as a bridge between mechanistic cell biology studies and phase 1 trials in human patients [17,18]. In most cases, laboratory animals such as the rabbit are used for initial studies because of their small size, low cost and faster progression of osteoarthritis. However, lapine (rabbit) cartilage is very thin, the tissue available for analysis is limited and this species retains intrinsic repair abilities at maturity [19,18,20]. Furthermore, smaller laboratory animals maintain a markedly flexed knee joint position at rest, whereas larger species have knee joint angles that are closer to that of the human knee [21]. It is therefore common that larger animals such as sheep, goats or horses are used to establish efficacy in models where serial synovial fluid analysis, topographical analysis of joint cartilage, and semi-invasive surgery are possible. It thus can be seen that *in vitro *and *in vivo *animal studies seldom use cartilage from the same species and anatomic location.

There are numerous cartilage repair treatments, including cell-based strategies such as the implantation of autologous chondrocytes (ACI) or engineered tissues [22]. ACI is performed with chondrocytes taken from a small biopsy, which are expanded *in vitro *[23]. Growth factors are added to chondrocyte cultures to prevent de-differentiation and to increase cell numbers [24,8,26]. It has been found that chondrocytes in the ankle and knee joints react differently to cytokine stimulation (IL-1) in rats and humans [27,28]. However, not much is known about the effect of growth factors on chondrocytes from different anatomical locations. In this study, two different growth factors were used in chondrocyte cultures: transforming growth factor β (TGFβ) and basic fibroblast growth factor (FGFb).

Transforming growth factor β (TGF-β1) is a pleiotropic cytokine that has many effects on chondrocytes. TGF-β1 can control cell proliferation, differentiation, and extracellular matrix (ECM) synthesis, as well as the biological activities of other growth factors [29]. Its effects on articular chondrocyte proliferation can be either stimulatory or inhibitory, depending on culture conditions, time of TGF-β1 addition to the culture, and state of cellular differentiation. Vivien *et al*. [30] and Fortier *et al*. [31] showed that TGF-β1 inhibited the growth of cells with 2% foetal calf serum (FCS), whereas TGF-β1 in media with 10% FCS caused a growth increase. Furthermore, the number, type and specificity of cytokine receptors, and their reaction to stimuli, may vary between joints. It has been shown that the size of the type II TGF-β receptor differs between freshly isolated and cultured bovine chondrocytes by 15 kD [32]. Also, Glansbeek *et al*. [2] found a species specific difference in chondrocyte expression of type II TGF-β receptor isoforms between murine, human and bovine cartilage. The murine cartilage taken from the patella expressed almost equal amounts of TGF-βbRII_1 _and TGF-βbRII_2 _mRNA, while human cartilage from femoral condyles expressed about three times more TGF-βbRII_1 _than TGF-βbRII_2_. In bovine articular cartilage from the metacarpophalangeal joint, only mRNA of TGF-βbRII_1 _was found. Studies have shown that in lapine chondrocytes, expression of TGF-β1 receptor systems is dependent on the stage of the cell cycle [30].

Basic fibroblast growth factor (FGFb) has been shown to prevent chondrocyte de-differentiation in culture [3] and decrease the doubling time of chondrocytes in monolayer cultures [33,25]. Chondrocytes, which have become fibroblast-like cells, are elongated and contain a distinct F-actin fibrillar structure. Martin *et al*. [3] concluded that chondrocytes treated with FGFb did not develop thick F-actin fibers, but instead had F-actin configurations resembling those of differentiated chondrocytes. The F-actin configuration is important since it influences the shape of the chondrocytes. FGFb was found to have a positive effect on lapine chondrocytes embedded in agarose grafts. These grafts produced better results in a modified histological O'Driscoll score compared to TGFβ or BMP treated agarose grafts [8].

This investigation studied the effect of species, anatomical location, and exogenous growth factors on chondrocyte proliferation *in vitro*. The large animal species and sites commonly used for harvesting chondrocytes chosen for this study were: the equine and ovine knee, shoulder and fetlock joints, as well as bovine fetlock joints. Equine cartilage was included in the study because the equine knee joint can be affected by naturally occurring disease. Furthermore, its thickness, which is similar to that of the human knee, allows for surgical manipulations [34,35]. The ovine knee is a common animal model used for cartilage repair studies [36,12]. The ovine shoulder joint provides more cartilage for *in vitro *studies than the fetlock joint and was also used as a third joint for comparison. The equine and ovine fetlock joints were compared to the bovine fetlock joint since it is commonly used for *in vitro *studies.

## Methods

### Chondrocytes and culture

Articular cartilage was harvested under sterile conditions from equine (EQ) and ovine (OV) shoulder (S), knee (K) and fetlock (F) joints, as well as from bovine (BO) fetlock joints within 24 h of death (Table 1). Animals between 1 – 8 years of age were available from the necropsy room or slaughterhouse, respectively. The animals had no joint or systemic disease (such as endotoxemia), which could affect chondrocyte metabolism. Cartilage samples from donors of different ages were not pooled.

**Table 1:** Distribution of sample collection

	Sheep (OV)	Horse (EQ)	Cattle (BO)
Knee (K)	2	5	0
Shoulder (S)	4	3	0
Fetlock (F)	3	3	3

The chondrocyte isolation protocol was the same in all species. Cartilage was removed from articular surfaces, washed with DMEM/F-12 (Invitrogen™, Burlington, Canada) containing 100 U penicillin, 100 μg streptomycin and 0.25 μg amphotericin B (Invitrogen™, Burlington, Canada), then minced into 1 × 2 mm pieces. The cartilage pieces were incubated with pronase E (Sigma, Oakville, Canada) (25 U/ml DMEM/F-12, and 8 ml/g cartilage) for 1.5 h at 37°C and 5% CO_2_, washed, and digested with bacterial collagenase type II (Sigma, Oakville, Canada)(400 U/ml DMEM/F-12 + 10 % foetal bovine serum (FBS)(Sigma, Oakville, Canada) and 8 ml/ g cartilage) for 3 h at 37°C and 5 % CO_2_. The cells were separated from undigested material through a sieve, centrifuged at 200 × g for 10 minutes to form a pellet, washed twice and resuspended with DMEM/F-12 + 10 % FBS. Cells were seeded at a density of 0.5 × 10^6^/ml (± 0.025 × 10^6^/ml) and 4 ml DMEM/F-12 + 10 % FBS per 25 cm^2 ^flask.

### Growth factors

After 2 days in culture, the media was changed and either 10 μg/ml recombinant human basic fibroblast growth factor (rhFGF-b) (R & D Systems, Inc., Minneapolis, USA), 5 μg/ml recombinant transforming growth factor β (rhTGF-β) (R & D Systems, Inc., Minneapolis, USA) or both factors were added twice every other day. Chondrocytes without added growth factors served as a control. The media in the control groups was changed at the same time as that in the growth factor treated groups. In each experiment, each group consisted of two flasks, except for chondrocytes from the ovine fetlocks. Due to the size of the ovine fetlock joint, fewer cells were collected, thus producing only one flask.

### Cell count

On day 6, the culture was 80–90% confluent and the chondrocytes were removed from all culture flasks by trypsinization, centrifuged at 200 × g for 10 minutes and washed twice with DMEM/F-12 medium. Day 6 was chosen for this procedure because the de-differentiation of chondrocytes, which has been observed in monolayer cultures, is still not very advanced at this stage [33,37]. The collagen type II to collagen type I ratio as an indicator for de-differentiation experienced the biggest changes in monolayer cultures between weeks 1 and 2 [37]. Before proceeding with further experiments, the influence of growth factors and cell culture conditions on the first passage was assessed. Cell count and cell viability were performed with the trypan blue exclusion test.

### Flow cytometry

**Figure 1:**
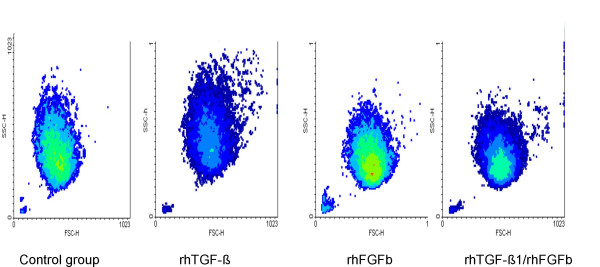
Density plots (FACS) of equine chondrocytes after 6 days in culture and/or two stimulations with growth factors (FSC: forward scatter (size); SSC: sideward scatter (granularity))

**Figure 2:**
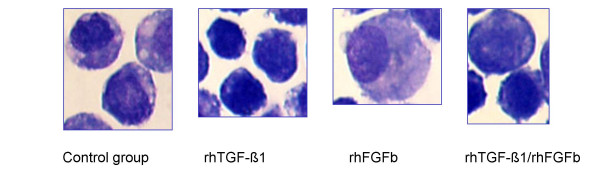
Cytospin slide preparation of equine chondrocytes stained with Wright's stain (Mag. 100×)

Flow cytometry analysis of fluorescent dye labelled chondrocytes (data not shown) stimulated with FGFb and TGF-β1 revealed an influence of the growth factors on cell size and granularity. The morphological profile of a cell can be observed by combining forward light scatter (FSC) and orthogonal or side light scatter (SSC). The FSC measurement is related to the cell size and can change through cell cycle progression and activation. Measurement of light scattered at a 90° angle, SSC, is related to internal granularity. Changes in these parameters were also found in the non-labelled cells and therefore flow cytometry analysis was performed in this investigation to study influence of growth factors on cell activation (Figure 1). Changes in cell morphology were confirmed with a cytospin slide preparation of the chondrocytes stained with Wright's stain (Figure 2). The cell size and granularity were analysed by flow cytometry with 0.5 × 10^6^/ml cells in serum-free DMEM/F-12. The analyses were performed with a FACScan (Becton Dickinson, Oakville, Canada) by gating 10,000 events. The settings were as follows: excitation: 488 nm; emission: 585 nm; forward side scatter (x - axis) – photodiode voltage set to E00, AmpGain set to 1.00, Lin; side scatter (y - axis) – photo multiplier voltage set to 410, AmpGain 1.12, Lin. The same settings were used throughout the whole experiment for all species and joints. The results of FSC and SSC measurements are given in the mean value of channels. A channel is a measured value of a parameter, representing the signal intensity of an event after amplification. To appear on a plot (FSC: x- axis; SSC: y-axis), data for an event must fall into one of 1024 channels. The mean value is calculated from 10,000-gated events. A conversion from the channel value to cell size in μm was not performed.

### Statistical analysis

A random block design was used in this study. The cell proliferation rate (increase in cell numbers), cell size and cell granularity of growth factor treated chondrocytes were compared to chondrocytes free of any treatment (control group). The results of the equine and ovine shoulder and knee joints were analysed separately from bovine, equine and ovine fetlock joints. A three factor ANOVA with paired Tukey's Correction was used for statistical analysis. The level of significance was p ≤ 0.05.

## Results

### Cell number

After 6 days in culture, cell numbers had increased slightly in control groups for EQ-F (equine fetlock), OV-S (ovine shoulder), OV-F (ovine fetlock) and BO-F (bovine fetlock) chondrocytes in contrast to EQ-K (equine knee), EQ-S (equine shoulder), and OV-K (ovine knee), where no increase in cell numbers was found. The cell viability was over 95 % similar to chondrocyte viability immediately after isolation. Chondrocyte monolayer cultures had variable proliferation profiles after the addition of FGFb and/or TGF- β. The addition of FGFb led to a significant increase in cell numbers for all species and joints (Table 2). The highest increase in cell count was found in BO-F, followed by EQ-F and OV-S chondrocytes cultures, as compared to the control groups. The addition of TGF-β increased cell numbers significantly in EQ-F (p = 0.0004) and in EQ-S (p = 0.0002) chondrocyte cultures, but showed nearly no mitogenic effect on EQ-K, OV-K, OV-S, OV-F and BO-F chondrocytes. Addition of both growth factors (FGFb and TGF-β1) to equine chondrocytes from all tested joints resulted in a higher increase in cell numbers compared to that from the addition of FGFb alone. In contrast to these findings, increases in cell numbers were lower in ovine and bovine chondrocyte cultures when both growth factors were added, compared to the addition of FGFb alone.

**Table 2:** Comparison of cell number, cell size and granularity of growth factors treated chondrocytes to control groups

**Cell number**	**FGFb**	**TGF-β1**	**FGFb/TGF-β1**
EQ-F	0.003 ↑	0.0004 ↑	< 0.0001 ↑
EQ-S	0.0031 ↑	0.0002 ↑	< 0.0001 ↑
EQ-K	0.1500	0.7494	0.0077 ↑
OV-F	0.016 ↑	0.667	0.153
OV-S	< 0.0001 ↑	0.8037	0.0204 ↑
OV-K	0.1223	0.4245	0.1249
BO-F	< 0.0001 ↑	0.964	< 0.0001 ↑
			
Cell size			
EQ-F	0.4983	0.4709	0.2509
EQ-S	0.8786	0.0061 ↓	0.2194
EQ-K	0.0033 ↑	0.4283	0.0008 ↑
OV-F	0.0343 ↓	0.4044	0.9033
OV-S	0.6132	< 0.0001 ↑	< 0.0001 ↑
OV-K	0.4310	0.0041 ↑	0.0041 ↑
BO-F	0.036 ↓	0.001 ↓	< 0.0001 ↓
			
Cell granularity			
EQ-F	0.0015 ↓	0.0514	0.0009 ↓
EQ-S	0.0074 ↓	0.2470	0.0020 ↓
EQ-K	0.0995	0.0098 ↑	0.0273 ↓
OV-F	< 0.0001 ↓	0.0996	< 0.0001 ↓
OV-S	0.0092 ↓	0.0005 ↑	0.1356
OV-K	0.0008 ↓	0.3767	0.0009 ↓
BO-F	< 0.0001 ↓	0.6367	< 0.0001 ↓

↑ : significant increase

↓ : significant decrease

Equine fetlock chondrocytes responded with the largest cell expansion to the addition of FGFb and FGFb/TGF-β whereas the knee chondrocytes expressed the lowest reaction to the growth factors. In the ovine species, the growth factors had the greatest effect on shoulder chondrocytes and fetlock chondrocytes, whereas knee chondrocytes responded with a lower proliferation rate. The bovine species showed the highest proliferation rate of all the tested species with respect to stimulation of fetlock chondrocytes with FGFb.

Cell proliferation rate of chondrocytes is age dependent. In this study, specimens from animals over 1 year of age were used and a trend of higher proliferation rates in younger animals was present, when compared to the older animals (up to 8 years of age). The influence of age on cell proliferation was significant, when data from animals < 1 year old were included (data not shown). Age did not influence the species and anatomical location differences or the chondrocyte reaction to growth factor treatment.

### Cell size

Chondrocyte cell size was given in mean value of channels and was affected by the addition of growth factors (Table 2). FGFb (p = 0.0033), and the combination of both factors (p = 0.0008) increased the cell size significantly in EQ-K chondrocytes. Equine shoulder chondrocytes were significantly smaller after treatment with TGF-β1, compared to control cells. In contrast to the equine species, the cell size in ovine shoulder and knee chondrocytes increased significantly after the addition of TGF-β1 to the culture, as compared to the control groups. The average ovine fetlock chondrocyte was significantly smaller after the treatment with FGFb (p = 0.0343). Combined treatment with both growth factors led to larger shoulder (p < 0.0001) and knee (p = 0.0041) chondrocytes in the ovine species. All treatments in bovine cultures resulted in significantly smaller chondrocytes (FGFb: p = 0.036; TGF-β1: p = 0.001; FGFb/TGF-β1: p < 0.0001). An interesting additional observation, without further investigation, was that the ovine chondrocytes were generally larger and more granular than the equine chondrocytes.

### Cell granularity

After the treatment with FGFb, all chondrocytes, except EQ-K, showed a significant decrease in cell granularity (Table 2). The same results were found for the combined treatment trials, except for the OV-S chondrocytes. The addition of TGF-β1 to the EQ-K and OV-S chondrocyte cultures resulted in an increase of granularity. Correlation between cell number, size and granularity was analysed using the Pearson Correlation Test and no relevant correlation was found.

## Discussion

Distinctions in cartilage, related to anatomical location (*i.e*. from knee and ankle joints), in regard to catabolism have been found [38]. Furthermore, the frequency of human osteoarthritis (OA) differs between joints. It is most common in the hands, knees and hips, while occurring less frequently in ankle and shoulder joints [16,38]. Dieppe and Kirwan [16] listed several factors that influence susceptibility and severity of osteoarthritis. Several authors have reported differences between joints [27,38], but this factor is not always considered when observations from *in vitro *studies are transferred to *in vivo *studies. This investigation details the influence of species and anatomical location on chondrocyte proliferation, as well as the influence of growth factors on chondrocytes from different joints and animals.

Isolated chondrocytes are used for cartilage repair in human patients [23] and in tissue engineering. These techniques rely on *in vitro *expansion of chondrocytes from small biopsies. The goal is to expand the chondrocytes quickly and to maintain their chondrogenic potential and their ability to re-differentiate. To this end, different growth factors and growth factor combinations have been tested [33]. However, the differences related to anatomical location of the knee and hip chondrocytes were not accounted for [33]. In tissue engineering, cells are placed in a three dimensional matrix to form new cartilage [39,40,25]. However, the newly formed tissue can vary from species to species, despite the fact that the chondrocytes were taken from the same anatomical location [39]. These studies involved the encapsulation of bovine and ovine chondrocytes into a photo-polymerising hydrogel system and it was found that bovine chondrocytes produced more glycosaminoglycans, whereas the cell number increased in the ovine constructs.

It was evident in our study that chondrocytes from the fetlock have a higher proliferation rate than chondrocytes from the knee joint in horses, as well as in sheep. This result indicates that the chondrocyte cell cycle varies according to species and location. The doubling time of human chondrocytes in monolayer cultures ranges between 1.7 to 3.5 days [23,33], while in equine and bovine chondrocytes it is 5 – 6 days [41,1]. In our study, doubling of the chondrocytes could not be achieved within the 6-day culture time for the control groups. It would be of interest to study several time points to evaluate changes over time and find the optimal time point for the addition of growth factors and their combination for chondrocyte expansion. When several time points are studied, the differentiation state of the cells has to be considered, because the de-differentiation progress in monolayer culture changes dramatically between the first and second week [37].

Age and culture conditions are also factors to consider when working with isolated chondrocytes. Age is an important factor, since young animals and young humans have chondrocytes that proliferate much faster than chondrocytes from older animals and humans [22,26]. The influence of age on the proliferation rate was observed to be significant when analysing data obtained from young animals (data not shown). In animals over 1 year of age, a trend of higher proliferation rates in younger animals was present, when compared to the older animals (up to 8 years), but age did not influence the species and anatomical location differences or the chondrocyte reaction to growth factor treatment. Therefore, in this study, only specimens from animals over 1 year of age were taken, because at this age their growth is no longer exponential [42]. Sheep, horses and cattle reach skeletal maturity at the age of 3–4 years [43,44]. Preliminary experiments showed that cell number could not be maintained at a steady state in the control group without the addition of FBS. Thus 10% FBS was added to the chondrocyte cultures. When chondrocytes are expanded for later *in vivo *use, they are cultured with homologous serum. This was not an option in this study because the specimens were retrieved from the necropsy room or slaughterhouse.

Chondrocytes modify their cell shape from round to flat in monolayer cultures and increase their DNA synthesis [45]. Lee *et al*. [1] suggest that these cell shape changes affect responsiveness to IGF-1 due to receptor level changes or affinity of the receptors in flattened cells. A change of cell size and granularity was found after cells were twice treated with growth factors FGFb and TGF-β1, which are indicators for different cell activation stages. Further investigations are necessary to study the influence of cell size and granularity changes on intracellular signalling. Changes in granularity can be caused by an engorged endoplasmatic reticulum, as a consequence of increased intracellular protein production.

It should be noted that human recombinant growth factors were also used in this study, since only bovine FGFb is available. The amino acid homology between human growth factors FGFb and TGF-β1 and the tested animals is above the 60 % threshold, which is necessary for cross-reaction between species [46]. Furthermore, FGFb and TGF- β1 belong to the β-sheet based folds family, which makes cross-reaction between species more likely, since these are highly conserved cytokines. The amino acid homology to human growth factors is 98% for equine, 97% for ovine and 98% for bovine FGFb and 84% for equine, 87% for ovine and 86% for bovine TGFβ-1. Increased biological activity may be more influenced by the receptor binding capacity and efficiency [46]. If the differences in cell proliferation were related to species-specific cytokine – receptor-binding interaction, the same proliferation pattern within the tested species would be found. It is very unlikely that the same growth factor interacts with cell surface receptors differently in different joints within one species. Rather, the inconsistent results between joints of the same species indicate the presence of different numbers of receptors on the cell surface.

The varying response between joints can also be due to different types or number of receptors per cell. Bovine fetlock chondrocytes express only one type of TGF-β receptor (TGF-βbRII_1_) in contrast to murine and human knee chondrocytes, which express two types of receptors (TGF-βbRII_1 _and TGF-βbRII_2_) [2]. The expression rate of the TGF-βbRII receptor is higher in freshly isolated bovine metacarpophalangeal chondrocytes as compared to cultured chondrocytes [47]. These findings indicate that time of treatment may influence receptor expressions. It would be of interest to study whether the changes in receptor density could be avoided by three dimensional chondrocyte cultures.

A synergistic effect of TGF-β1 and FGFb is reported in the proliferation rate of human hip and ankle [33], as well as, in lapine knee chondrocytes [48]. In our study, the same effect was found on equine chondrocytes from all tested joints. In contrast, ovine chondrocytes from the various joints and the bovine fetlock chondrocytes did not demonstrate this effect. The proliferation rate in ovine and bovine chondrocyte cultures with the addition of both growth factors was lower than the cultures with FGFb alone. This synergistic effect is species specific, but is not related to the anatomical location of cartilage origin.

The signal pathways activated by growth factors are complex. TGF-β1 activates two independent signal pathways, which lead to the activation of different transcription factors [49]. It could be speculated that the smaller cell size is a result of freshly divided cells, but a correlation between cell number and size was not found in our study. To investigate the cause of the cell size and granularity changes after growth factor treatment, further studies such as cell cycle studies and/or intracellular matrix protein production studies would be necessary. It would also be of interest to analyse the type and number of FGFb and TGF-β receptors on the cell surface of the chondrocytes from different joints and their changes over culture time. A different number of cell surface receptors on chondrocytes may suggest that chondrocytes from one joint produce more matrix than chondrocytes from another joint. This factor would be an important consideration when harvesting chondrocytes for tissue engineering. For example, a smaller cartilage biopsy from the ankle joint would be necessary to produce the same amount of new tissue than a bigger biopsy from the knee joint [38].

Healthy chondrocytes from various joints within the same species proliferate differently, depending on the addition or absence of growth factors. This indicates varying capabilities of articular chondrocytes to react to stimuli, which is further supported by the catabolic differences found between human ankle and knee cartilage explants [38]. Therefore, one could expect that an *in vitro *study performed with fetlock chondrocytes regarding cell proliferation and tissue engineering gives different results when knee chondrocytes are used. Furthermore, the use of a different species could add another variable. The use of animal models to establish treatment strategies for osteoarthritis before clinical studies is very important since technical application difficulties and the development of associated joint pathology would be revealed in these models [22,20].

## Conclusion

The animal model, site and the size of the lesion require careful consideration in making decisions about treatment strategy [22]. *In vitro *studies are important to adequately preparing for *in vivo *testing. Thus, *in vitro *studies should be performed using the species and site where subsequent *in vivo *studies are planned.

## Competing interests

The author(s) declare that they have no competing interests.

## Authors' contributions

MKA made substantial contributions to conception and design as well as acquisition and interpretation of data.

MBH revised the article critically and gave final approval of the version to be published.

## Pre-publication history

The pre-publication history for this paper can be accessed here:


